# A method for the ultrastructural preservation of tiny percutaneous needle biopsy material from skeletal muscle

**DOI:** 10.3892/ijmm.2013.1454

**Published:** 2013-07-23

**Authors:** TIZIANA PIETRANGELO, STEFANO PERNI, GUGLIELMO DI TANO, GIORGIO FANÒ-ILLIC, CLARA FRANZINI-ARMSTRONG

**Affiliations:** 1 Department of Neuroscience and Imaging, Section of Physiology and Physiopathology, ‘G. d’Annunzio’ University of Chieti-Pescara and Clinical Research Center (CRC) of G. d'Annunzio Foundation, Chieti I-66100, Italy; 2 Department of Cell and Developmental Biology, University of Pennsylvania School of Medicine, Philadelphia, PA 19104, USA

**Keywords:** light and electron microscopy, muscle biopsy, muscle ultrastructure, tissue micromanipulation

## Abstract

Skeletal muscle biopsies require transecting the muscle fibers resulting, in structural damage near the cut ends. Classically, the optimal ultrastructural preservation has been obtained by the use of relatively large biopsies in which the tissue fibers are restrained by ligating to a suitable retaining support prior to excision, and by examining regions at some distance from the cut ends. However, these methods require invasive surgical procedures. In the present study, we present and substantiate an alternative approach that allows for the excellent ultrastructural preservation of needle biopsy samples, even the very small samples obtained through tiny percutaneous needle biopsy (TPNB). TPNB represents an advantage, relative to standard muscle biopsy techniques and to other needle biopsies currently in use, as in addition to not requiring a skin incision, it leaves no scars in the muscle and requires an extremely brief recovery period. It is most appropriate for obtaining repeated samples in horizontal studies, e.g., in order to follow changes with athletic training and/or aging in a single individual and for studies of sarcopenic muscles in elderly patients. Due to the small size of the sample, TPNB may present limited usefulness for classical pathology diagnostics. However, it offers the major advantage of allowing multiple samples within a single session and this may be useful under specific circumstances.

## Introduction

We recently described a procedure for human muscle biopsy that we termed tiny percutaneous needle biopsy (TPNB) and we demonstrated that it is an excellent method for obtaining human skeletal muscle specimens with the least trauma for the patient (1). The classically and commonly used needle biopsy technique is needle aspiration biopsy (NAB), using a Bergstrom needle (2,3). Similar to NAB, TPNB involves a percutaneous approach (thus no need for an invasive skin incision) and the use of a penetrating needle. However, TPNB differs significantly in the considerably smaller size of the sample and in the more automated and rapid penetration of the needle into the depths of the muscle, resulting in a considerably less traumatic approach and relatively rapid and orderly regeneration of the tissue (1). To support the low invasiveness of TPNB, we previously analyzed nuclear magnetic resonance (NMR) images of muscles following TPNB (unpublished data). At this low level of resolution, the muscle showed no apparent traces of lesions or wounding. This evidence has convinced us that TPNB has great potential for use in human muscle studies, particularly in horizontal studies, requiring repeated samples from the same subject, i.e., before and after a specific stimulus, such as a training period. The positive aspects of this less invasive method may prove to be advantageous when the volunteers are medium-high level athletes.

Although only a few milligrams of muscle are collected using TPNB, the quality of these specimens allows their use in a large variety of cellular and molecular approaches, including cell culture, functional studies of single dissociated muscle fibers and RNA and protein analysis in transcriptional and proteomic studies (4–6). However, the issue of whether the very small biopsy samples can be used for structural and/or ultrastructural analysis, remains unresolved. The cutting of the muscle fibers with a blade that is part of both open surgery and needle biopsy procedures, promotes the immediate depolarization of the fibers, and hence their contraction. The fibers, however, relax after a very brief period and if, as in a well-managed open biopsy (7), they are restrained by ligating to a suitable retaining support prior to excision, they maintain their resting length and alignment. In a needle biopsy of any size, the fibers are detached from the surrounding muscle tissue and after the initial contraction they will remain at a shorter length as the connective tissue will impede their passive lengthening to the initial resting length. Additional considerable disarrangement ensues as bundles of fibers within the sample are variously oriented. To avoid these major drawbacks of needle biopsy for structural studies, we explored the possibility that, if properly handled, an excellent structural preservation of the small sample could be obtained. The procedure involves the transferring of the section of muscle from a needle biopsy to a solution that mimics the intracellular medium (high potassium, low calcium) and keeps the fibers depolarized and thus not excitable, and in the appropriate sample micromanipulation in order to allow restoration to the initial ‘resting’ sarcomere length or close to it. To that effect, using light microscopy and thin section electron microscopy, we analyzed muscle samples obtained from mice both by standard dissection and by TPNB, as well as samples from human subjects obtained by TPNB. The aim of this study was to clearly demonstrate that good ultrastructural preservation can be obtained from TPNB samples.

## Materials and methods

**Figure 1:**
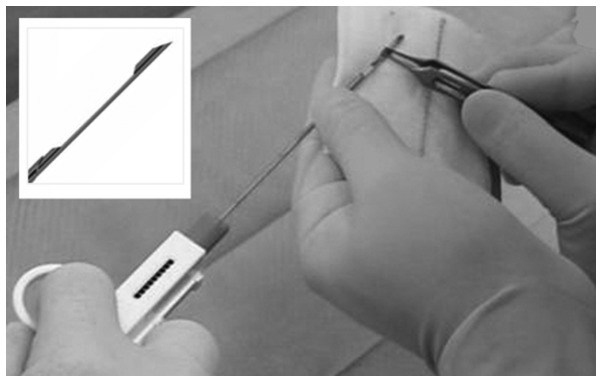
Semiautomatic needle biopsy device for TPNB. Shown is the semiautomatic Tru-cut type system for performing multiple biopsies on skeletal muscle. The external cutting cannula has been removed in order to take the muscle specimen. The insert (upper left corner) shows the notch, 20 mm in length, that traps muscle on the needle. The cutting cannula (not shown) are designed to provide the sharpness required to obtain a clean, uncrushed specimen. Repositioning the needle into the external cannula allows for subsequent samples to be obtained.

Muscles from euthanized mice (under IACUC protocol approved by the University of Pennsylvania) were used in two different sets of experiments. One was in determining the effects of two different high potassium solutions on the ultrastructure of intact muscle fibers, and the second in devising the best approach for the preservation of needle biopsy material. First, the extensor digitorum longus (EDL) muscles were carefully dissected, pinned to a Sylgard dish, exposed to a solution that replaced extracellular sodium with potassium and chelated extracellular calcium. Two different solutions were used: either a ‘KCl’ solution [150 mM KCl, 5 mM MgCl_2_, 3 mM ethylene glycol tetraacetic acid (EGTA), 10 mM phosphate buffer, pH 7.2] or a ‘K acetate’ solution (150 mM K acetate, 5 mM MgSO_4_, 10 mM EGTA, 10 mM phosphate buffer, pH 7.2). Muscles were kept for ~3 min at room temperature in one of these two solutions and then fixed in freshly prepared 3.5% glutaraldehyde in 0.1 M cacodylate buffer, pH 7.2 for ~1 h. Secondly, using a small size needle (semiautomatic 14-gauge needle biopsy device: Vantage GS, Zamar S.r.l., Suzzara, Italy; Precisa 1310, HS, Precisa Medico Corp., Latina, Italy) (Fig. 1) biopsies were taken from the gastrocnemius and the masseter. The latter muscle was more suitable as the needle was relatively large for the leg muscle of the mouse. The samples that were expelled directly from the needle into the ‘K acetate’ solution at room temperature contained several small coherent bundles of fibers with different orientations and the fibers appeared mostly wavy. Individual bundles of parallel fibers were separated by gentle teasing, straightened out by ‘combing’ and/or stretching out with tweezers or syringe needles while viewing under a dissecting microscope and then immersed in fixative solution as described above. The fixed bundles were stored at 4°C, for different periods of time.

We further obtained three small biopsy samples from the vastus lateralis (VL) muscle from 32–66-year-old male healthy volunteers (indicated as M32, 32 years old; M33, 41 years old; M34, 66 years old, respectively) using the TPNB procedure as described in our previous study [Pietrangelo *et al*(1)]. The study was approved by the Ethics Committee of the University of Chieti-Pescara (approval protocol no. 1233/06 COET). Each subject provided written informed consent. Biopsy was performed under local anesthesia using 2 ml carbocaine (20 mg/ml; AstraZeneca S.P.A., Basiglio, Italy) following skin sterilization with betadine. We used semiautomatic needle biopsy devices as for the mouse, but 13-gauge in size. The cylinder of the muscle thus obtained had a cross-sectional area of ~3 mm^2^ and a length of ~4 mm. Despite the small needle diameter, the TPNB VL muscle specimens were of adequate size and good quality. The specimens were immediately immersed in ‘K acetate’ solution and then treated as for the mouse.

All fixed muscles were further rinsed in buffer, post-fixed in buffered 2% osmium tetroxide (OsO_4_), and the block was then stained in saturated uranyl acetate and embedded in Epon 812. Sections (~40 nm thick) were cut using a Leica Ultracut R microtome (Leica Microsystems, Vienna, Austria) using a Diatome diamond knife (Diatome Ltd., Biel, Switzerland) and stained with lead citrate solution. The sections were imaged in using a Phillips 410 electron microscope (Philips Electron Optics, Mahwak, NJ, USA) with a Hamamatsu C4742-95 digital camera (Advanced Microscopy Techniques, Chazy, NY, USA).

Some fibers were separated from the mouse K-acetate treated biopsy bundles following osmium fixation. After gentle teasing to separate them, the fibers were whole-mounted in 100% glycerol under a coverslip and imaged using a Nikon microscope equipped with phase contrast optics and a Nikon Digital Sight DS-Fi1 camera.

The canonical number of three individuals for the human samples is sufficient to assess the quality of structural preservation based on the criteria shown.

## Results

### Effects of extracellular K and Cl on muscle ultrastructure

**Figure 2:**
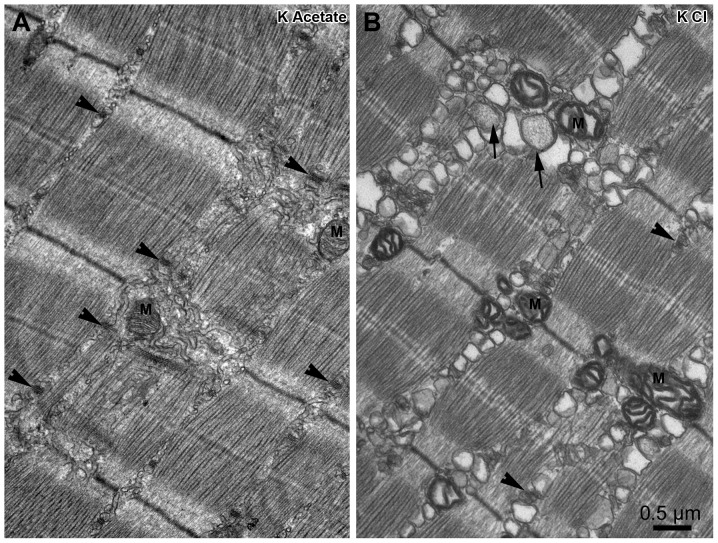
Intact mouse extensor digitorum longus (EDL) muscle exposed either to (A) 150 mM ‘K acetate’ or (B) 150 mM ‘KCl’ solution. (A) ‘K acetate’ shows excellent ultrastructure preservation. The arrowheads indicate the triads located at the A–I junctions. The sarcoplasmic reticulum and tubules are in the form of a continuous network. Mitochondria (M) have a compact structure. The cross striation is orderly and there are no excessive empty spaces between the myofibrils. (B) ‘KCl’ illustrates the swelling effect of Cl^−^ and H_2_O inflow. The sarcoplasmic reticulum network is fragmented into large empty vacuoles. The arrows indicate modified calsequestrin-filled cisternae that appear enlarged into spherical sacs. The arrowheads indicate altered triads, still partially identifiable. Mitochondria (M) appear having dilated cristae. Despite these modifications, the cross striation remains well aligned.

The initial immersion of the small biopsy samples in a ‘high potassium’ balanced salt solution requires some caution, as it is known that the exposure of a cell (or muscle fiber) to a solution in which sodium has been replaced by potassium, instantaneously depolarizes the cell and subsequent to depolarization, the cell will take up permeant anions (chloride ions if present) through the sarcolemma from the extracellular space, thereby swelling (8). Thus, it is important to substitute the extracellular space anion with one that is not permeable through the plasmalemma (e.g., acetate). Fig. 2 graphically illustrates the effects of exposing a whole, undamaged mouse muscle to a solution containing 150 mM of either K acetate (Fig. 2A) or KCl (Fig. 2B). In the K acetate solution, the muscle fibers have the classical well preserved structural appearance. Triads (arrowheads) are located at the A–I junctions, the sarcoplasmic reticulum (SR) elements in between are in the form of a continuous network (reticulum) constituted of elongated tubules, with slightly wavy but mostly longitudinal orientations. The mitochondria (M) have a compact structure. The cross striation is orderly and there are no excessive empty spaces between the myofibrils. By contrast, Fig. 2B, shows the damaging effects of exposure to a high KCl solution. The most obvious effect is the swelling and vacuolization of the SR; the whole network is fragmented into large empty vacuoles. Some triads (arrowheads) are still partially identifiable, but the calsequestrin-filled cisternae are often enlarged into spherical sacs (short arrows). The mitochondria (M) have dilated cristae, and the normal close proximity between the mitochondria and triads (9) is markedly affected. Large empty spaces are also created between the myofibrils (data not shown). Despite these changes, the cross striation remains well aligned.

### Orderly structure of muscle fibers from TPNB biopsy at the light microscope level

**Figure 3:**
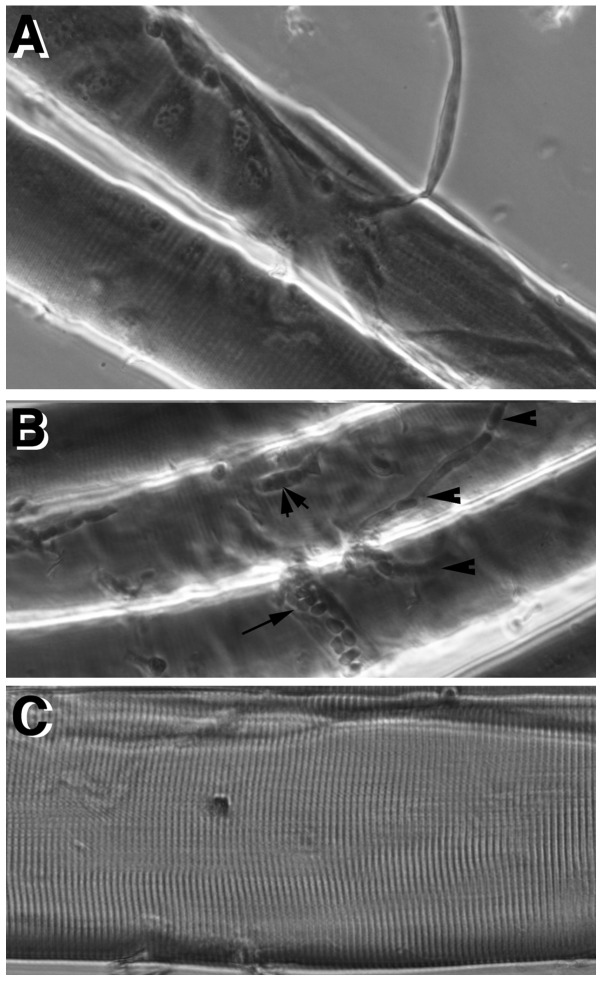
Light microscopic images of mouse skeletal muscle fibers obtained by TPNB. The procedure preserves the fiber organization and some of the accessory component of the muscle. (A) Terminal branches of nerve endings in proximity of neuromuscular junction in the masseter muscle. (B) Some small blood vessels (arrowheads) and a big vessel with blood cells (arrow), together with a satellite cell (double arrowhead), are still visible on the masseter fiber surface. (C) Gastrocnemius muscle fiber showing the regular pattern of striation without contractures.

If appropriately handled, as described in Materials and methods, the small biopsy specimens show long stretches of fibers that maintain excellent order, with no indication of distortions or contractures. This is shown in whole mount preparations of fibers teased from fixed mouse biopsies and observed under phase contrast optics, as described in the study by Boncompagni *et al*(10). Fig. 3A shows terminal branches of nerve endings in proximity of neuromuscular synapses; Fig. 3B illustrates small capillaries (arrowheads), a slightly larger blood vessel filled with blood cells (small arrow) and a satellite cell (double arrow-head) over the fiber surface. Noticeably, cross striation has a very regular spacing (barely visible in Fig. 3A and B, but clearly visible in Fig. 3C).

### Optimum preservation of muscle ultrastructure is possible in TPNB samples

**Figure 4:**
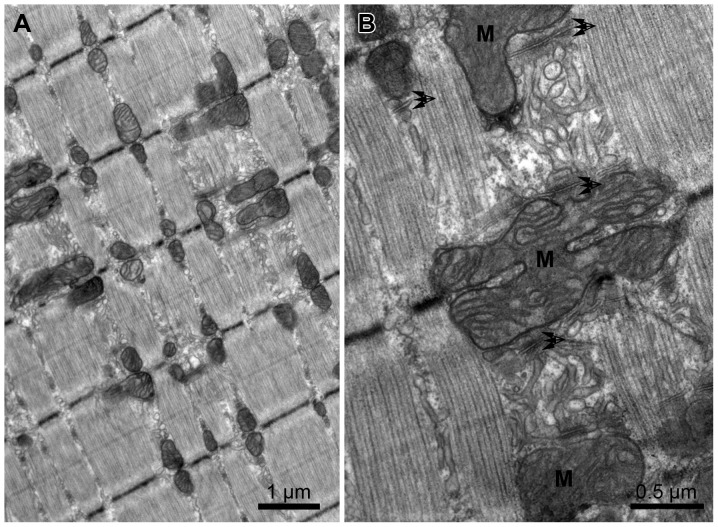
Ultrastructure of a sample obtained by TPNB from a mouse masseter muscle. (A) Low magnification image showing the preservation of the overall muscle structure in a longitudinal section. No contraction of the fiber is noted, triads and mitochondria are organized in close proximity. (B) High magnification image showing a well defined I band, aligned sarcomere components and a good preservation of the sarcoplasmic reticulum (SR) compartments. The triads lay at the edge of A band (triple arrows) and the lateral sacs of the SR are located in close proximity to the mitochondria (M).

Successful electron microscopy depends on the appropriate orientation of the muscle fibers in the embedding and this was obtained by our technique of selecting small ordered bundles of fibers from the center of the biopsy sample, and improving their orientation by gently ‘combing’ and mechanically stretching the bundle. The image of the mouse biopsy samples shown in Fig. 4A confirms well aligned cross striations and an orderly arrangement of the mitochondria, observed as dense structures aligned on either side of the Z line.

At higher magnification (Fig. 4B) the ordered sarcomeres show a well defined I band and a sharp edge of the A band. Triads (triple arrows) are well positioned and show the appropriate association between the central T tubules and the two SR lateral sacs. Most important is the fact that the continuity of the SR network and the detailed positioning of its longitudinal tubules, fenestrated collar opposite the centre of the A band and terminal cisternae at the triads are well preserved. This is clearly visible where the SR is cut tangentially near the mitochondria. The close association between the mitochondria and the lateral sacs of the triad [Boncompagni *et al*(9)] is maintained. Fragmentation and vesiculation of the SR of the type shown in Fig. 2 is not present in the central regions of the biopsy bundle treated with K acetate, although these alterations of course occur very close to the cut ends.

### Biopsy samples obtained from human subjects by TPNB

**Figure 5:**
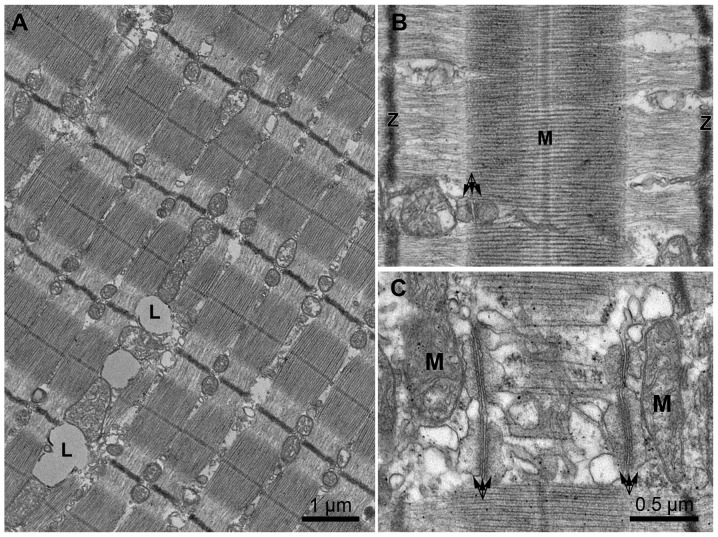
Ultrastructure of a sample from a human vastus lateralis (VL) muscle obtained by TPNB discarded from the needle into a ‘K acetate’ solution. (A) Low magnification image of the regular striation pattern of the fibers, no sign of local contractures and lipid droplets (L). (B) Details of sarcomeres as well aligned bands, a straight Z line and a prominent M line (M). (C) Architecture of the membrane components with the appropriate filament orientation at the edges of the A band (triple arrows indicate a triad) and the mitochondria (M) appropriately positioned. (A and B) Donor M34 (66 years old); (C) donor M32 (32 years old).

The TPNB human biopsy samples shown in Fig. 5 were discarded from the needle into a ‘K acetate’ solution and treated as the mouse biopsies. Low magnification images of TPNB samples (Fig. 5A) show the regular striation pattern of the fibers and no sign of local contractures as already evinced by light microscopy, although the final sarcomere length varied between 2.3 and 3.0 μm in the various samples. This is due to different amounts of stretching during the preparation step, prior to fixation, a good indication that the fibers were relaxed at that point. An advantage of this approach is that the amount of stretching applied to the small muscle bundles can be modulated by the operator during the pre-fixation step.

The details of the sarcomeres confirm optimal preservation, showing well aligned bands and well preserved sarcomere details, with aligned filaments, a fairly straight Z line and a prominent M line (Fig. 5B). The architecture of the membrane components is retained: the triads (Fig. 5C, arrows) have the appropriate orientation at the edges of the A band and the mitochondria (M) are appropriately positioned near them. The SR is well differentiated into terminal cisternae, filled with calsequestrin, and a tubular longitudinal SR segment. The empty spaces between the SR elements contain glycogen granules (data not shown), lost during the post-fixation protocol.

## Discussion

In this study, we describe a simple procedure for preserving the ultrastructural details and for appropriately orienting the muscle fibers for electron microscopy observation within muscle bundles obtained by small needle muscle biopsy (TPNB procedure). We focused on TPNB as it presents an ultimate challenge to ultrastructural preservation due to the very small size of the sample. The procedure that we devised can of course be applied to standard needle biopsy samples obtained with larger size needles. It was generally considered that short damaged segments of fibers fixed without restraint would be liable to be distorted and thus unusable for analysis. The novelty of our procedure is in keeping the fibers in the biopsy sample quiescent by depolarizing them in a high potassium solution and in using careful micromanipulation in order to obtain small, but well oriented bundles of fibers that are straight and stretched to various sarcomere lengths. We also used EGTA to chelate extracellular calcium, thus decreasing the contracting effect of the prolonged depolarization. Due to the slow diffusion of calcium within the muscle cytoplasm (0.014 μm^2^/msec) (11) we do not expect entry of extracellular calcium from the cut ends to induce contractures within a few microns from the cut surface.

TPNB is a relatively easy, rapid and inexpensive method of obtaining samples using semiautomatic needles developed for clinical investigations of other organs; however, thus far it has not been implemented for basic human muscle research. If used for muscle biopsies, the procedure that we developed depends on a strict collaboration between the surgeon and an electron microscopist who has access to a good dissecting microscope within a very short period of time and is capable of fine micromanipulations. Two important considerations are that the small bundles of fibers to be fixed and embedded should be well oriented and that the solution used for depolarizing the fibers should contain an impermeant anion in place of Cl^−^ in order to avoid fiber swelling. If a fairly immediate means of dissection is not available, the biopsy samples should be immersed in the high K acetate solution and aerated until ready for use. A prolonged delay is not advisable.

The value of the TPNB procedure lies not only in its feasibility, but also in its minimal invasiveness, which makes it particularly useful for vertical studies of muscle adaptation, such as in athletes. In addition, we visualize the technique as being particularly appropriate for the sampling of sarcopenic skeletal muscle of elderly subjects. Sarcopenia is a condition that is defined by progressive atrophy of the skeletal muscle (12), although it is not yet fully understood, particularly in terms of the ultrastructural adaptation. This has largely been due to the difficulties in obtaining samples from fragile subjects, such as the elderly and in pathological conditions resulting in severe muscle atrophy where the sampling has to be reduced as much as possible. However, the usefulness of the procedure is limited in the case of some pathological conditions where large spatial variations in the distribution of connective tissue and fat exist, so that individual small biopsyies may collect little muscle tissue. The problem can be solved by collecting several samples, taking advantage of the minimal trauma involved in each, and using echographic imaging.

## Abbreviations

EGTA: ethylene glycol tetraacetic acid
NAB: needle aspiration biopsy
NMR: nuclear magnetic resonance
SR: sarcoplasmic reticulum
TPNB: tiny percutaneous needle biopsy
VL: vastus lateralis
